# Venous sinus injuries are common with occipital skull fractures

**DOI:** 10.1186/1757-7241-22-S1-O8

**Published:** 2014-07-07

**Authors:** Susan Hendrickson, Srinivas Murahari, John Scotter, Eilka Kashef, Bryn Jones, Mark Wilson

**Affiliations:** 1 London's Air Ambulance, London, UK; 2 Pan London Neurotrauma Group, London, UK

## Introduction

Traumatic cerebral venous sinus injuries are usually managed conservatively, however sinus thrombosis and obstruction can result in refractory intracranial hypertension.

## Methods

We retrospectively analysed CT venograms performed on 29 patients who had sustained a skull fracture that crossed a venous sinus at a London Major Trauma Centre.

## Results

**Figure 1:**
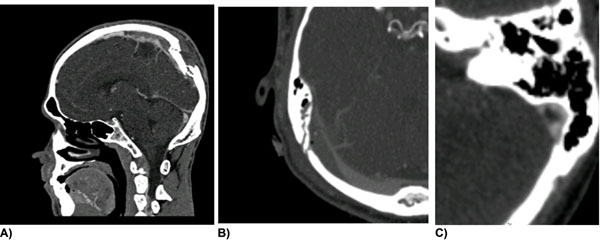
**A)** Displaced fracture causeing sagittal sinus thrombosis; **B)** Periosteal haematoma compressing right transverse sinus; **C)** Right Sigmoid Sinus thrombus forming under undisplaced right occipital / base of skull fracture.

18 of the 29 patients studied had either venous sinus thrombosis (14) or significant sinus caliber compromise (+/- thrombosis). Three mechanisms of sinus injury were noted in this group (Figure 1). A) Displaced fracture restricting sinus caliber; B) periosteal or extradural haematoma compressing the sinus and c) reactionary sinus thrombosis to an undisplaced overlying fracture.

## Conclusions

CT Venography should be considered in patients with fractures overlying a venous sinus especially in cases with refractory or disproportionate intracranial hypertension or headache out of keeping with imaging appearances. We demonstrate different types of injury and management options.

